# From Robot to Virtual Doppelganger: Impact of Visual Fidelity of Avatars Controlled in Third-Person Perspective on Embodiment and Behavior in Immersive Virtual Environments

**DOI:** 10.3389/frobt.2019.00008

**Published:** 2019-02-18

**Authors:** Geoffrey Gorisse, Olivier Christmann, Samory Houzangbe, Simon Richir

**Affiliations:** LAMPA, Arts et Métiers ParisTech, Présence et Innovation, Angers, France

**Keywords:** virtual reality, embodiment, avatar, doppelganger, Proteus effect

## Abstract

This study presents the second phase of a series of experiments investigating the impact of avatar visual fidelity on the sense of embodiment and users' behavior in immersive virtual environments. Our main focus concerns the similarity between users and avatars, a factor known as truthfulness. Our experiment requires the participants to control three avatars using a third-person perspective: a robot, a suit and their virtual doppelganger (virtual representation of the self). In order to analyze users' reactions and strategies, each task of the scenario of the virtual reality application can potentially affect the integrity of their characters. Our results revealed that ownership, one of the three factors of the sense of embodiment, is higher for the participants controlling their self-representation than with abstract representations. Furthermore, avatar visual fidelity seems to affect users' subjective experience, half of the panel reported having different behavior depending on the controlled character. Abstract representations allow the users to adopt more risky behaviors, while self-representations maintain a connection with the real world and encourage users to preserve the integrity of their avatar.

## 1. Introduction

One of the major challenges concerning virtual reality studies is to determine the predominant factors and parameters influencing user experience in immersive virtual environments (Cummings and Bailenson, 2016). In this context the relation between users and avatars has been the focus of recent researches from different disciplinary fields. We designed a series of experiments to investigate the effect of avatar visual fidelity on user experience in immersive virtual environments. Our aim is to analyze the impact of controlling different characters, including our own representation modeled through a 3D reconstruction process, in terms of embodiment (Kilteni et al., 2012a) and behavior.

The goal of the previous and first phase of our series of experiments was to investigate the impact of truthfulness (similarity between the user and the virtual character, Garau, 2003) and self-esteem on perceived attractiveness as well as character selection (Gorisse et al., 2018). The participants were immersed in front of three humanoid characters presenting a constant level of realism (consistent with the proposed virtual environment) and three different levels of visual fidelity: a robot (**Figure 2A**), a suit (**Figure 2B**) and a virtual doppelganger (virtual version of the self, Bailenson and Segovia, 2010) (**Figure 2C**). Due to the high realism and truthfulness levels induced by the 3d reconstruction of the users, we also considered the possible occurrence of the Uncanny Valley phenomenon (Mori et al., 2012). Indeed, in the context of our experiments, avatars' truthfulness and realism levels can potentially induce revulsion responses toward the 3D models if inconsistencies are observed by the participants on their virtual self-representation (Seyama and Nagayama, 2007; MacDorman et al., 2009).

Our results demonstrate that attractiveness evaluations are significantly higher for the virtual doppelgangers (direct integration of the participants' heads enabled by 3D scanning technologies) and highlight the impact of self-esteem on character selection, especially between the extreme conditions (robot vs. virtual doppelganger). The higher the self-esteem scores, the more the participants are prone to choose their own representation and the lower the self-esteem scores, the more they favor abstract characters. These results underline the potential benefits of proposing personalized avatars, especially based on users' appearance. However, the interviews revealed interesting insights. Some participants agreed that, depending on the context of each virtual reality experience, abstract characters allow to increase disconnection from reality.

Based on these results, the second phase of our experiment reported in this article investigates the impact of avatar visual fidelity in terms of embodiment and behavior, a phenomenon known as the Proteus effect (Yee and Bailenson, 2007; Yee et al., 2009). Previous work demonstrates that facing a virtual doppelganger in immersive environments can impact users' attitudes (Fox and Bailenson, 2009; Bailenson and Segovia, 2010; Aymerich-Franch et al., 2014), but few studies have investigated the impact of controlling in real-time one's own representation via a full-body tracking system (visuomotor synchrony). Nonetheless, the work of Waltemate et al. (2018) provides evidences concerning the impact of avatar personalization on the sense of embodiment in virtual environment using the classical mirror metaphor and a first-person perspective. In this context, our study aims to extend such previous results to potential new use cases relying on a third-person perspective enabling to perceive the whole body of the controlled avatar (Gorisse et al., 2017). Thus, in this study, the participants embody three characters presenting different levels of truthfulness (**Figure 2**) and are involved in a series of tasks and situations that may potentially affect their avatars' integrity in order to investigate the effect of avatar visual fidelity on the sense of embodiment and on users' behavior in immersive virtual environments.

The next section presents a state of the art concerning avatar visual fidelity, the sense of embodiment and the behavioral implications induced by avatars' appearance. Section 3 presents the experiment and its protocol. Results are reported in section 4 and are discussed in section 5. Section 6 concludes the study before the introduction of our future work in section 7.

## 2. Related Work

### 2.1. Avatar Visual Fidelity

Visual fidelity has been classified by previous work into three criteria (Garau, 2003; Mansour et al., 2006):

- Anthropomorphism (*non-humanoid - humanoid*).
- Realism (*few detailed - photorealistic*).
- Truthfulness (*does not look like the user - looks like the user*).

**Anthropomorphism** corresponds to the morphological characteristics of the virtual character. The Cyborg dilemma theory underlines the potential influence of avatar virtual morphology on users' body perception: “*Choose technological embodiment to amplify the body, but beware that your body schema and identity may adapt to this cyborg form.”* (Biocca, 1997). Thus, avatar morphological inconsistencies would lead to a distortion of the users' mental model who will adapt their body schema to that of the controlled avatar. However, recent studies demonstrate the adaptation capacity of human brain. Thus, the concept of homuncular flexibility (Won et al., 2015) illustrates this ability to control additional or abnormal limbs (Kilteni et al., 2012b; Steptoe et al., 2013; Hoyet et al., 2016; Laha et al., 2016).

In the frame of our study, we use humanoid avatars controlled in real time thanks to a full body tracking system. Indeed, congruent visuomotor synchrony implies similar body schema between users and avatars to enable a natural transfer of our navigation and interaction mechanisms.

**Realism**, especially perceptual (McMahan, 2003), corresponds to the level of detail of meshes and textures of 3D models. If in the past it was mainly limited by hardware constraints related to the computing power required for real-time 3D rendering, it is now possible to create realistic and optimized models. However, according to Zell et al. (2015) it seems that realism is not a good predictor for attractiveness. This study also illustrates the importance of consistency between the stylization level of characters' shapes and materials. Indeed, inconsistencies negatively impact appeal and attractiveness of virtual characters making them eerie. Fleming et al. (2017) study also illustrates that shapes and proportions significantly influence attractiveness evaluations of virtual characters. Furthermore, it has been demonstrated that being confronted to a realistic autonomous agent can induce social behaviors, such as shyness or shame, by influencing the perception of the personality and social characteristics of virtual characters (Volante et al., 2016).

It should be noted that while the level of perceptual realism seems to influence users' subjective experience, it can also impacts performance, particularly in terms of interaction. For instance, the work of Argelaguet et al. (2016) demonstrates that controlling an abstract virtual hand induces a higher sense of agency than a realistic hand while allowing faster and more precise interactions.

**Truthfulness** is the degree of similarity between users' appearance and virtual characters (Benford et al., 1995, 1997). This dimension of visual fidelity constitutes the heart of our study, 3D reconstruction of users being the highest reachable truthfulness level.

Previous research on doppelganger (virtual version of the self) (Bailenson and Segovia, 2010) highlights an impact on users' behavior when the latter are confronted to their own representation. It is demonstrated that users do not respect the same social norms especially concerning interpersonal distances when facing their own representation (Bailenson et al., 2001, 2008). Indeed, it seems that virtual version of the self can be processed by the brain in a similar way to real self (Gonzalez-Franco et al., 2016). Thus, studies demonstrated some potential benefits of observing our doppelganger such as a higher involvement in sports tasks after vicarious reinforcement (Fox and Bailenson, 2009) or preparation for public speaking (Aymerich-Franch and Bailenson, 2014).

The work of Lucas et al. (2016) on video-games and of Waltemate et al. (2018) on immersive environments, respectively, demonstrate a better user engagement and a higher sense of ownership with virtual self-representation. However, little is known concerning the impact of controlling in real-time our doppelganger perceived from a third-person perspective in terms of embodiment and behavior in immersive virtual environments.

As our study focuses on the impact of truthfulness and involves avatars with a high degree of realism, the Uncanny Valley phenomenon has to be considered. This theory suggests that a person's response to a robot, or by extension to a virtual character, could shift from empathy to revulsion if the highly realistic model try to achieve a lifelike appearance but presents inconsistencies (Mori et al., 2012). Previous work highlights different factors influencing 3D models perception such as textures, shading or shapes (MacDorman et al., 2009; Zell et al., 2015). Moreover, virtual characters' animation amplifies the acceptance or revulsion potential (McDonnell, 2012). Thus, one of the most important factor contributing to acceptance and plausibility of 3D characters is therefore the coherence between appearance, animation and behavior (Volante et al., 2016). While we are able to a use motion capture systems to control virtual characters' bodies in real time, Latoschik et al. (2016) pointed out the importance of facial animation. Nonetheless, current immersive setups relying on virtual reality headsets do not allow to track facial expressions. Implementation of such technologies should be investigated in a near future to allow for richer non verbal interaction methods, especially for multi-user studies.

**Figure 1:**
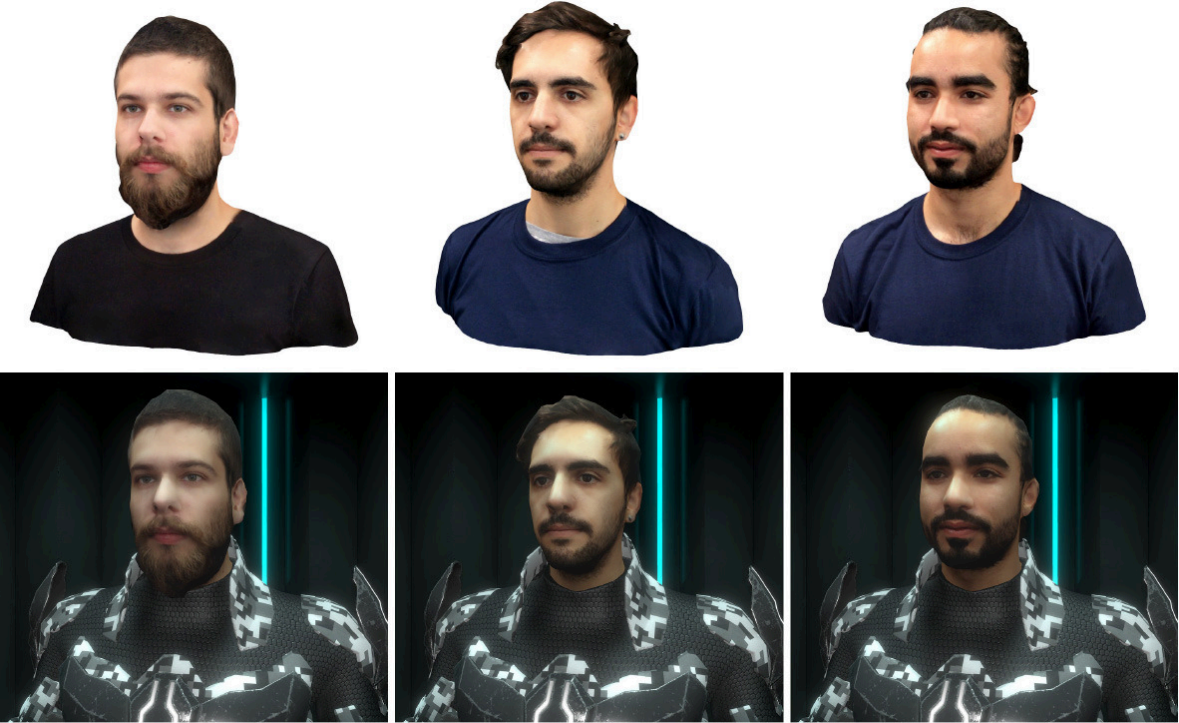
Illustration of three raw bust models (50K polygons) and of their optimized versions (2.5K polygons) integrated in the virtual reality application. Written informed consent was obtained from the depicted individuals for the publication of this image.

In the context of our experiment, it is also necessary to consider current hardware limitations concerning 3D scanning technologies. While it is not the main focus of our experiment, the results of the previous phase of our study revealed that some participants (*N* = 6) seem to be disturbed by the presence of minor issues suggesting the occurrence of the Uncanny Valley phenomenon (Gorisse et al., 2018). Indeed, due to the realism and truthfulness levels of the scanned characters (Figure 1), participants have high expectations and are more prone to notice avatars' flaws (Seyama and Nagayama, 2007; Lugrin et al., 2015). Furthermore, suspension of disbelief is harder to reach, users' virtual self-representation maintaining a link to the real world.

### 2.2. Embodiment

The sense of embodiment refers to the feeling of being located inside, of owning and controlling another body. Although it is not possible to physically dissociate ourselves from our biological body nor to occult stimuli from the real world, it was nevertheless demonstrated the possibility of biasing our body perception. The initial paradigm of the rubber hand illusion (RHI) proved that it was possible to generate a proprioceptive drift from a real hand toward a fake one using synchronous visuotactile stimulations (Botvinick et al., 1998). This experiment induces a mislocalization and a sense of ownership of the fake limb.

Embodiment in a virtual environment arouses the interest of communities of researchers studying different media including video games (Taylor, 2002) and virtual reality (Normand et al., 2011; Kilteni et al., 2012a; Spanlang et al., 2014). As part of our reflection, we will adopt the working definition proposed by Kilteni et al. (2012a):

“*The sense of embodiment toward a body B is the sense that emerges when B's properties are processed as if they were the properties of one's own biological body”*(Kilteni et al., 2012a).

In this framework, three dimensions allow the emergence and the sustainability of the sense of embodiment in virtual environments:

**The sense of self-location**, corresponding to a determinate volume in space where users feel located. Several phenomena can affect this sense of location. Previous experiments on out of body experiences demonstrated that congruent visuotactile stimulations induce a localization conflict (Ehrsson, 2007; Lenggenhager et al., 2007). The manipulation consists to confront the subjects with their own image thanks to a head-mounted display and a camera located behind them. Visuotactile stimulations are then repeated on the subjects' body, triggering a sensory conflict between visual perception and tactile information. Some experiences also demonstrate that it is possible to adopt a virtual agent's viewpoint during a face-to-face confrontation thanks to different mental projection strategies (Thirioux et al., 2009).

Furthermore, the perspective used in virtual worlds induced by the position of the camera also seems to be a determining factor on self-location (Slater et al., 2010; Petkova et al., 2011; Debarba et al., 2015; Gorisse et al., 2017). While some results diverge, recent work demonstrates that multisensory integration allow to embody a virtual avatar using both first and third-person perspective (**Figure 3**) but with significant differences concerning the self-location dimension (Debarba et al., 2017; Gorisse et al., 2017).

**The sense of agency** is defined by Blanke and Metzinger (2009) as the “*global motor control, including the subjective experience of action, control, intention, motor selection and the conscious experience of will"*. To induce a sense of agency, a correlation between the intention of the subject and the resulting action in the virtual environment is necessary. For instance, it was demonstrated that visuomotor synchrony induce a high sense of agency over virtual avatars (Debarba et al., 2015; Gorisse et al., 2017) or robotic hands (Caspar et al., 2015). Indeed, in this context the subjects are able to predict the outcomes of their movements in the virtual environment thanks to the natural navigation and interaction process afforded by the motion-capture system. However, while visuomotor synchrony appears to be very effective, it is demonstrated that action attribution leading to expected outcomes could be enough to induce a sense of agency (Kokkinara et al., 2016).

The recent study of Jeunet et al. (2018) investigates further the concept of agency in virtual environments thanks to brain computer interfaces and revealed that agency could be divided in two components, namely the feeling and the judgment of agency relying on three principles : priority, consistency and exclusivity.

**The sense of ownership**, also referred as one's self-attribution of a body (Kilteni et al., 2012a), was initially observed through the RHI paradigm (Botvinick et al., 1998). If it was assessed thanks to a similar experiment as for self-location, it remains a different factor of the embodiment process (Maselli and Slater, 2014). The adaptation of this paradigm in virtual environments revealed the possibility of experiencing a sense of ownership toward a virtual body (Slater et al., 2008). Indeed, multisensory integration appears as a critical factor allowing the emergence of the sense of embodiment in immersive environments to embody avatars with different morphological and demographic characteristics (Slater et al., 2010; Banakou et al., 2013; Kilteni et al., 2013; Peck et al., 2013). Here, bottom-up factors like visuotactile (Slater et al., 2009; Normand et al., 2011; Maselli and Slater, 2013; Kokkinara and Slater, 2014) and visuomotor synchrony (Sanchez-Vives et al., 2010; Kokkinara and Slater, 2014) are critical contributors to induce a sense of ownership toward a virtual body.

On the other hand, if it is acknowledged that top-down factors like morphological similarities and realism level are not mandatory to embody virtual characters, they nevertheless seem to positively impact the sense of ownership (Argelaguet et al., 2016; Latoschik et al., 2017). Moreover, the recent work of Waltemate et al. (2018) demonstrates that avatar personalization, based on users' real appearance, also induces a higher the sense of ownership in virtual environments. This study relies on the virtual mirror metaphor and first-person perspective, the latter being known to induce a high sense of embodiment (Slater et al., 2010; Maselli and Slater, 2013). In this context, our study aims to pursue the investigations concerning the impact of truthfulness on the sense of embodiment and users' behavior in a more complex scenario relying on third-person perspective (Debarba et al., 2015; Gorisse et al., 2017) to extend such previous results to potential new use cases.

### 2.3. Character Appearance and Behavior

Appearance of embodied virtual characters can affect users' attitude and behavior in virtual environments, as illustrated by the Proteus effect theory (Yee and Bailenson, 2007; Peña et al., 2009). This effect is based on two main theoretical foundations:

- Self perception theory: inference of attitudes and beliefs from self-observation.
- Deindividuation: decrease in self-awareness induced by anonymity or the fact to be in a group.

According to Yee and Bailenson: “*Users who are deindividuated in online environments may adhere to a new identity that is inferred from their avatars. […] users in online environments may conform to the expectations and stereotypes of the identity of their avatars.” (Yee et al*., *2009**)*

The implications of the Proteus effect in classic real-time 3D environments or immersive environments are multiple. It was initially demonstrated that avatar size and attractiveness can influence users' behavior and social interactions in immersive applications (Yee and Bailenson, 2007). Many studies on virtual body ownership highlighted the impact of virtual characters' properties such as skin color (Kilteni et al., 2013; Peck et al., 2013), age (Banakou et al., 2013), or gender (Slater et al., 2010) on participants' attitudes. Recent work aslo highlights the influence of avatars' appearance on the emergence of common fears in immersive environments such as acrophobia (Lugrin et al., 2016). Here, anthropomorphism influences the feeling of security when users are exposed to high elevation.

While virtual character appearance can impact users' behavior, it has also been shown that the embodiment of characters with strong cultural connotations can have a cognitive impact. Thus, the work of Osimo et al. (2015) shows that embodying an avatar representing Sigmund Freud induces enough detachment to allow the participants to change their usual ways of thinking. In the same context, the research of Banakou et al. (2018) shows that embodying Albert Einstein leads to improved performance when performing cognitive tasks. The literature review proposed by Hudson and Hurter (2016) also suggests that, in a learning context, avatar appearance could impact learners' attitudes and motivation.

In these different use cases, deindividuation can be used as a trigger increasing the effects of self-perception theory and its influence on users' behavior. However, in the context of our experiment, using doppelgangers will not lead to deindividuation due to the lack of anonymity.

Our literature review illustrates the plurality of factors that can influence the sense of embodiment as well as the ability of avatars to affect users' behavior. We propose to pursue investigations in this direction in order to identify the impact of avatar visual fidelity, especially truthfulness, on the sense of embodiment and its effect on users' behavior in immersive virtual environment.

## 3. Materials and Methods

**Figure 2:**
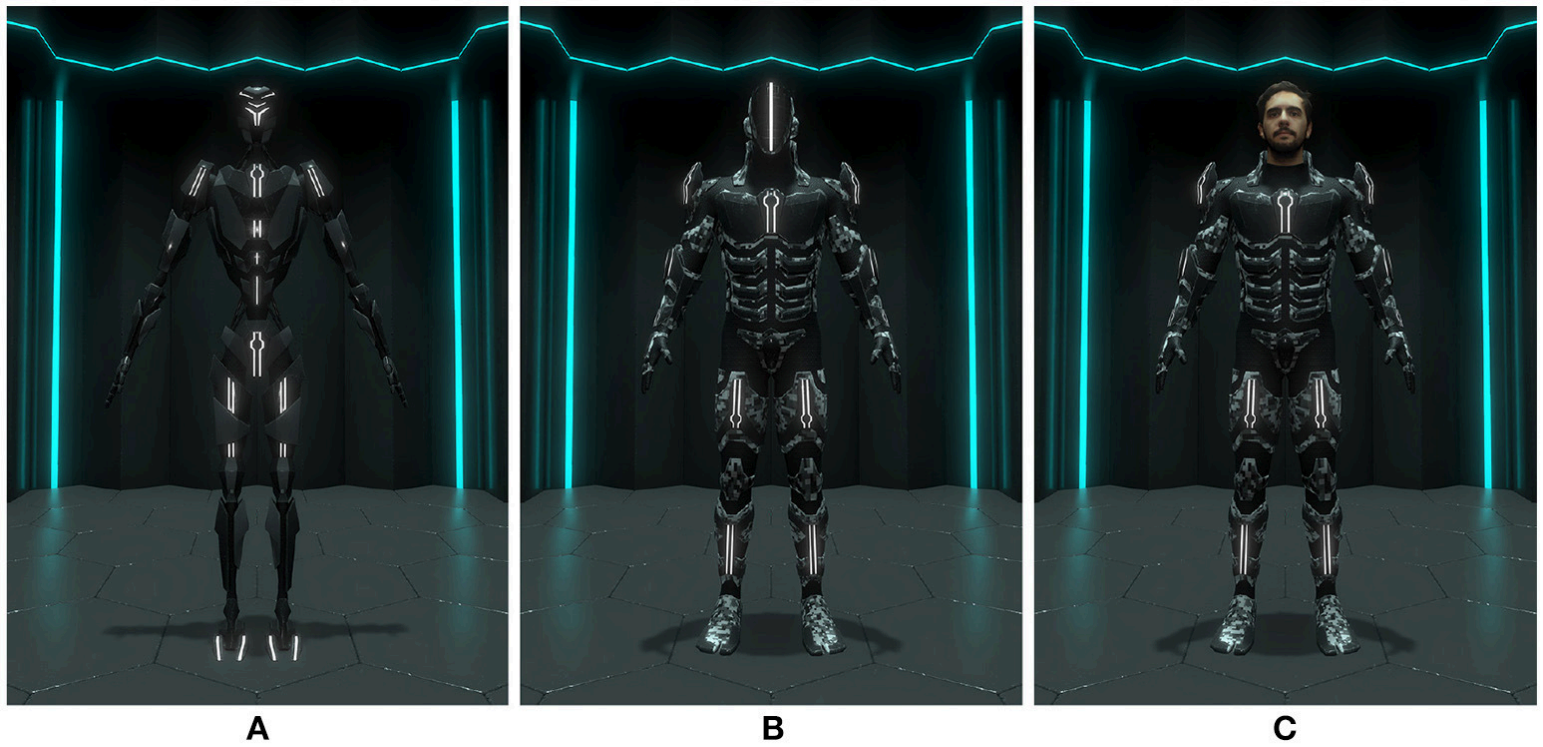
Screenshots of the three avatars presented to the participants in the virtual environment: **(A)** robot, **(B)** suit, **(C)** doppelganger. Written informed consent was obtained from the depicted individual for the publication of this image.

In order to investigate the impact of avatar visual fidelity, the participants are immersed and control three virtual characters (Figure 2) via a motion capture system. Each avatar presents a different truthfulness level (similarity between the avatars and the participants, Figure 1) increasing from a robot to virtual doppelgangers modeled via a 3D reconstruction process. However, each character presents a constant anthropomorphism level (humanoid body schema) and a realism level consistent with the virtual environment (Figure 2).

**Figure 3:**
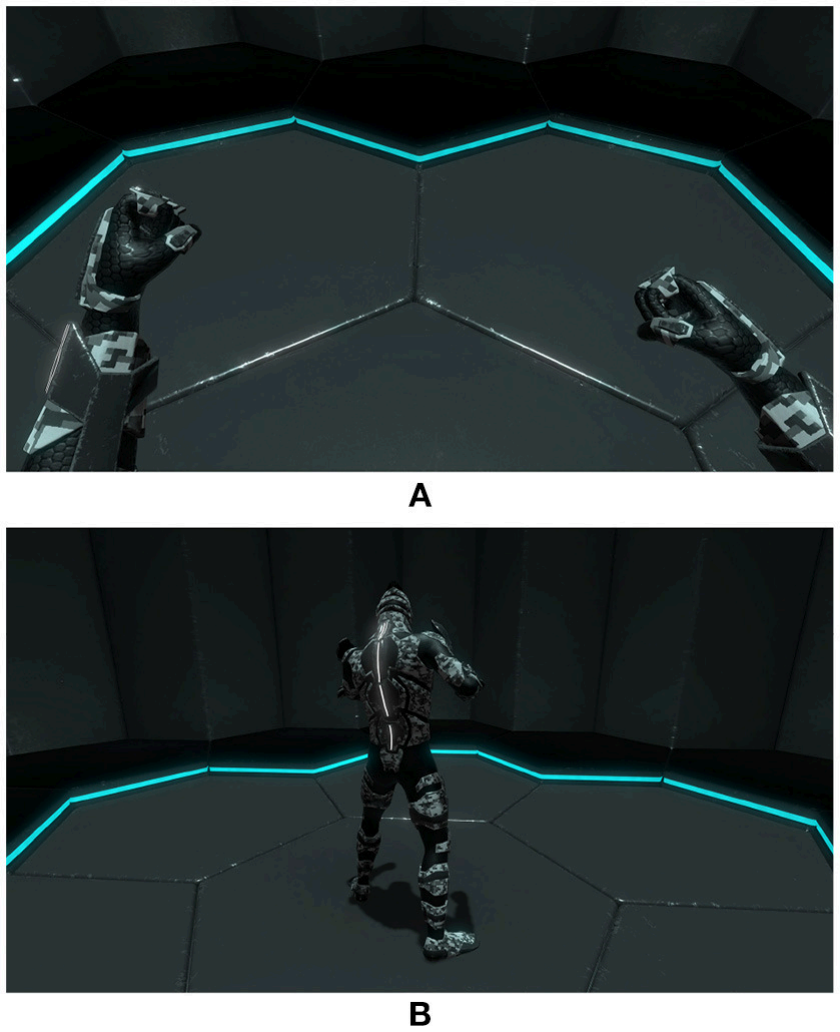
First- and third-person perspectives: **(A)** 1PP, **(B)** 3PP.

The scenario of the application developed for the experiment consists of an appropriation phase and five tasks (**Figure 5**) described in the next sections. Each task is designed to potentially affect the avatars' integrity, making it possible to verify the occurrence of the Proteus effect through the analysis of the participants' behavior and reactions. While many studies on embodiment in virtual environments are based on first-person perspective (Figure 3A) and real-time mirrors, the third-person perspective (Figure 3B) used in this experiment allows to perceive the whole embodied avatar while opening new use cases for virtual reality. Furthermore, previous work demonstrated that the sense of embodiment could be induced in third person perspective under synchronous visuomotor conditions (Debarba et al., 2015, 2017; Gorisse et al., 2017).

The sense of embodiment is subjectively assessed through a questionnaire and post-experiment interviews. The impact of avatar appearance on users' behavior is evaluated using both objective and subjective approach based on the collected data, the post-experiment questionnaire and the interviews.

### 3.1. VR Application

The content of the application was developed using the real-time 3D engine Unity. The choice of the graphic universe and the scenario legitimates and makes plausible the presence of the three avatars and their respective level of truthfulness (Figure 2):

- Robot (Figure 2A): the anthropomorphism level of the robot allows users to project their body schema to control it. However, its morphology does not allow them to imagine that they can be physically located "inside" the character as some parts of the robot (neck, abdomen, etc.) are designed not to match human size.
- Suit (Figure 2B): the suit is morphologically congruent with human size, potentially allowing users to imagine a projection of their body inside of it.
- Doppelganger (Figure 2C): the doppelganger is also scaled to human size and integrates the 3D reconstruction of the participant's head.

The artistic direction ensures the plausibility of the robot as well as the suit, because they appear coherent in this science fiction universe, which would not be the case in a contemporary environment. Concerning the doppelgangers, we consider that the current popular culture (video games, films, novels, etc.) allows users to accept the presence of human-like avatars in futuristic environments. This environment consists of a training room dedicated to the appropriation phase and a main arena constituted of modular hexagonal platforms enabling the place to be dynamically configured for each task. The lighting system is designed to maximize the affordance and allows the user to quickly distinguish neutral elements from hostile ones.

To prevent revulsion responses induced by this kind of environment, participants were asked to rate their level of affinity with science fiction thanks to a scale ranging from one to five in the pre-experiment questionnaire. Results indicate the high affinity of the panel with the proposed universe (*M* = 4.47, *SD* = 0.79). If it could be argued that such a rich environment could affect our results, it was nevertheless designed to maximize users' involvement in the different tasks of our experiment, which seems necessary to study user experience and behavior.

We implemented the third-person perspective based on previous work (Gorisse et al., 2017). The virtual camera is placed three meters behind the avatar (Figure 3B). Elements intersecting the field of view become transparent to avoid occlusion problems. The rotation of the camera is centered around the character and proportional to the rotation the participants' head. The full body tracking system enables the participants to navigate freely and naturally inside the virtual environment thanks to the real time synchronization of their whole body movements. While it requires a period of time to get accustomed to this new way of perceiving the controlled body, every participant mastered this interaction process in few minutes during the dedicated phase of the experiment, as described in the subsequent presentation of the protocol (section 3.4.1).

### 3.2. Apparatus

**Figure 4:**
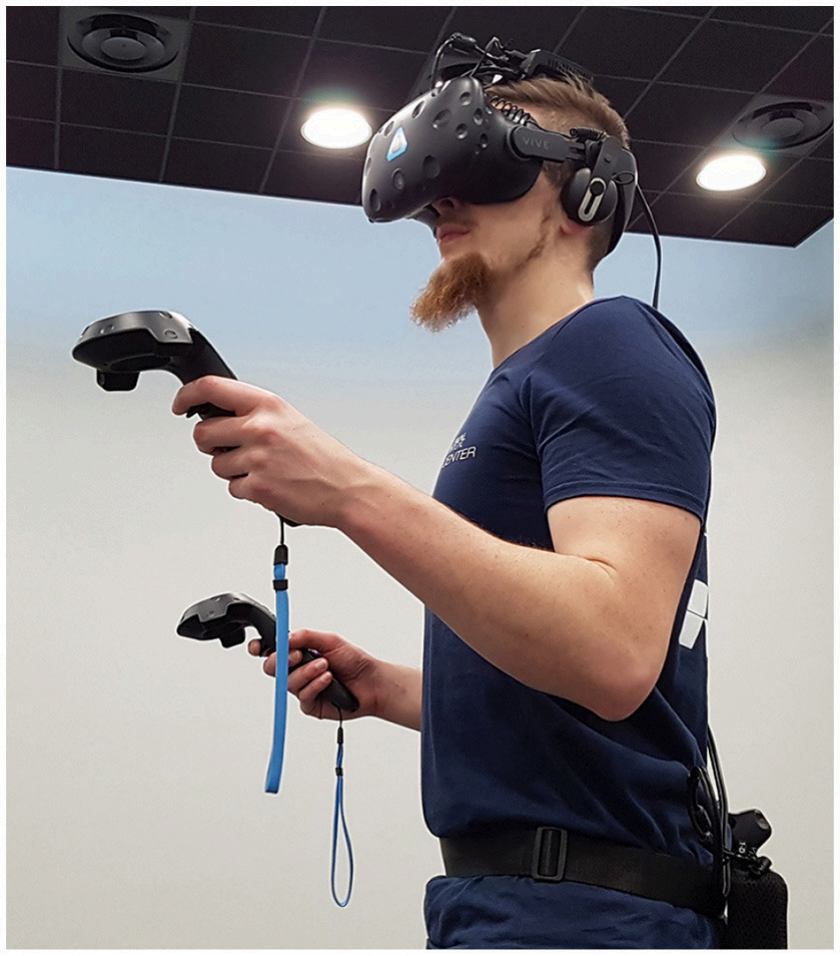
User equipped with the required devices for the experiment: HTC Vive virtual reality headset, Vive trackers, TP Cast wireless adapter. Written informed consent was obtained from the depicted individual for the publication of this image.

The HTC Vive virtual reality headset and a TP Cast wireless adapter (latency < 2 ms) are used to display the virtual environment with the following specs: a resolution of 2,160 × 1,200 pixels (1,080 × 1,200 pixels per eye), a field of view of 110° and a refresh rate of 90 Hz. We also use two Vive controllers and three Vive Trackers to track the body movements using inverse kinematics algorithms to animate the avatars in real-time (Figure 4). The computer running the application is composed of an Intel Xeon E5-1607 @ 3,10 GHz processor and a Nvidia GeForce GTX 1080 graphics card.

We measured the overall latency of the system using the manual frame counting method described in He et al. (2000). Thanks to a high-speed camera (240 Hz), we recorded simultaneously five hand-clapping motions by removing the lenses of the HTC Vive to be able to compare the delay between the real movement and its virtual counterpart displayed on the headset's screen. Analyzing the motions frame by frame, we measured an average shift of 3 frames (12.5 ms). Thus, our system benefits from these low latency devices providing an overall delay under the threshold of 20 ms recommended for virtual reality (Raaen and Kjellmo, 2015).

### 3.3. Participants

We recruited 34 male participants, aged from 21 to 42 (*M* = 23.79, *SD* = 3.80). As we experienced some issues concerning 3D reconstruction of long hairs, we are currently working on a complementary study to investigate the gender impact on the sense embodiment in virtual environments. Each subject has a correct or corrected vision thanks to glasses.

All the participants have used an immersive virtual reality system at least once and are experienced with video games involving the control of avatars (role playing games, first and third-person shooter, platformer, etc.). Two reasons led to the recruitment of experienced participants. First, it limits the duration of the appropriation process, this way the participants stay focused on the content of the experiment and not on the novelty of the devices. Second, virtual reality is currently reaching the mass market and we suppose that, in few years, most of the population will have access to this technology making our results more generalizable.

### 3.4. Procedure

At first, the subjects participated in a 3D scan session. Each 3D model was then optimized to be integrated in the application dedicated to the experiment (Figure 1). They were invited, a few days later, to participate in the experiment. After completing a consent form, they filled the pre-experiment questionnaire to collect their demographic information as well as their experience of video-game and virtual reality. Then, they were geared up with the equipment required for the experiment and received the starting instructions.

According to our within-subject design, the participants perform the scenario three times to control each avatar in a counterbalanced order. Between each session of the experiment, the participants complete a questionnaire. After passing the three conditions, a semi-directive interview is conduced to discuss about their subjective feeling and their experience with the different avatars.

#### 3.4.1. Tasks Description

The goal of the five tasks consist in activating a terminal always appearing on the opposite side of the virtual arena. Once triggered, the arena starts its configuration process to dynamically adapt the environment for the next task. Each task is designed to potentially affect the avatars' integrity. This punitive model encourages participants to perform the best of their possibility and allows to observe different behaviors and strategies.

**Figure 5:**
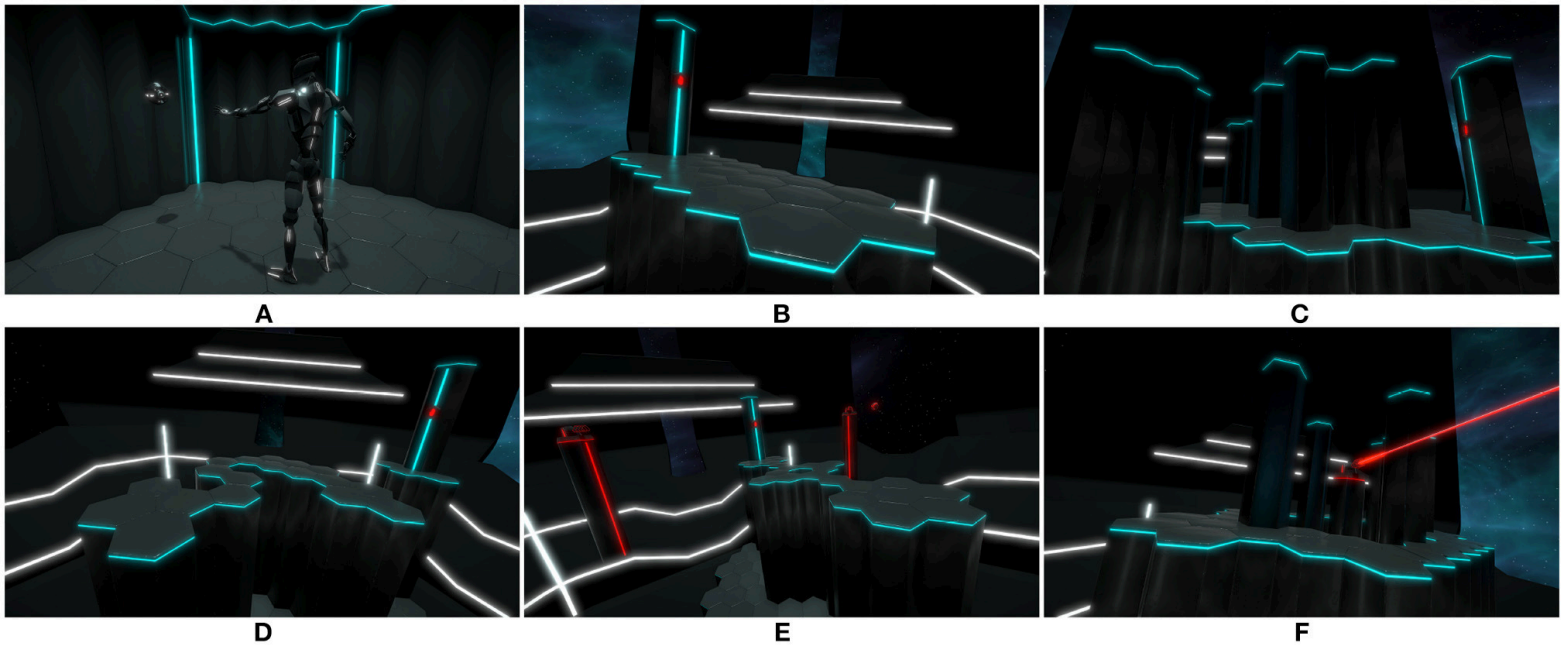
Virtual arena during the tasks of the experiment: **(A)** appropriation phase, **(B)** task 1: bridge, **(C)** task 2: walls and ledges, **(D)** task 3: traps, **(E)** task 4: spheres, **(F)** task 5: laser.

The experiment begins with the appropriation phase enabling the participants to calibrate their avatar. First, they can observe and turn around the avatar freely before they enter inside the virtual body to enable the calibration process in order to set the avatar's size and to activate the full body tracking. When calibrated, a drone enters the room and asks the user to reach its position five times. It should be noted that most of the instructions are explicated by the drone within the virtual environment to provide the same information for all the participants. During this appropriation phase, the participants are allowed to navigate within the first room until they feel at ease with the third-person perspective and their virtual body. Thus, when asked for cyber-sickness during the post experiment interview, every subject reported being comfortable thanks to this habituation process. After this first phase, they reach the central elevator to enter the arena to pursue the five tasks of the experiment (Figure 5).

**Task 1 - bridge:** The first task simply consists in crossing a bridge to activate a terminal enabling the configuration of the platforms for the second task.

**Task 2 - walls and ledges:** The second task requires the users to walk on ledges between walls and the void while being careful not to fall down.

**Task 3 - traps:** The third task is composed of a path with trapped platforms. Each platform can trigger the next section of the path or suddenly remove one of the next elements. The participants have to walk slowly to avoid being trapped by the moving platforms.

**Task 4 - spheres:** The fourth task requires the subject to cross the arena while dodging or blocking spheres shot by two turrets disposed on their left and right sides. The projectiles can only be blocked with the avatars' arms. If the participant is collided by two spheres, the avatar is switched in ragdoll mode and respawns after a fade out on a previous safe platform.

**Task 5 - laser:** During the last phase, the users face a rotating laser. At the beginning of this phase, they are protected by a pillar. They have to move or crouch to dodge the laser in order to reach the next pillars and the opposite side of the arena. If the participant is hit by the laser, the avatar is switched in ragdoll mode and begins the respawn process.

### 3.5. Measures

We based our measures on subjective (qualitative and quantitative) and objective data to collect and cross information concerning the sense of embodiment and users' behavior. After each of the three sessions of the experiment (one by avatar condition), a questionnaire is filled by the participants according to the procedure presented in the previous subsection. It consists of five-point semantic scale items divided into two dimensions (Table 1):

- Embodiment, consisting of ten items divided in three factors: self-location (SL), sense of agency (A) and sense of ownership (O). These items are based on previous work (Debarba et al., 2015; Argelaguet et al., 2016; Gorisse et al., 2017).
- Proteus effect, composed of two items assessing to what extent the participants feel vulnerable during the different tasks of the experiment depending on the controlled avatar.

**Table 1:** Post experiment questionnaire composed of three dimensions: embodiment (SL, Self-Location; A, Agency; O, Ownership; PE, Proteus effect).

**ID**	**Questions**
SL-Q1	To what extent did you feel that you were located inside the virtual body?
SL-Q2 (r)	To what extent did you feel that you were located at a certain distance from the virtual body, as if you were looking at someone else?
A-Q1	To what extent did you feel that the virtual body moved just like you wanted it to, as if it was obeying your will?
A-Q2	To what extent did you feel that the virtual body reacted in the same way as your own body?
A-Q3	To what extent did you feel that you were able to interact with the virtual environment the way you wanted to?
A-Q4	To what extent did you feel that you controlled the virtual body as if it was your own body?
O-Q1	To what extent did you feel that the virtual body was your own body?
O-Q2 (r)	To what extent did you feel that the virtual body was someone else's?
O-Q3	To what extent did you have the impression that when something affected the virtual body, your actual body was affected too?
O-Q4	To what extent did you forget your actual body in favor of the virtual body?
PE-Q1	To what extent did you feel vulnerable during the situations and events of the scenario (altitude, projectiles, laser…)?
PE-Q2	To what extent did you have the impression that the situations and events of the scenario (altitude, projectiles, laser…) could affect the integrity of your character?

Objective performance data are collected via the application in a CSV (*Comma-Separated Values*) file. This file contains the following information:

- Navigation duration (ND): completion time for each task of the scenario. It corresponds to the duration between the activation of the traveled section and the activation of the next one.
- Falls (F): number of falls during the whole experiment.
- Collided Spheres (CS): number of collided spheres during the task 4 (Figure 5E).
- Deviated Spheres (DS): number of deviated spheres during the task 4 (Figure 5E).
- Collided Lasers (CL): number of collisions with the laser during the task 5 (Figure 5F).

The experiment process is concluded with a semi-structured interview allowing the participants to express themselves on their experience. The questions address the sense of embodiment, as well as attitudes and strategies of the participants toward the situations that could potentially threaten their avatar. In other words, it helps to assess the occurrence of behavioral reactions induced by the avatars' appearance. The transcript of these interviews makes it possible to carry out a semantic analysis to identify recurring remarks and to gather some relevant statements in the frame of our research.

### 3.6. Hypotheses

H1 : Avatar truthfulness impacts positively the sense of embodiment, especially ownership, in immersive virtual environments.

H2 : Avatar truthfulness affects users' behavior and reactions toward situations that could potentially threaten their avatar.

## 4. Results

The Shapiro-Wilk Test was carried out to check the normality of the distributions of the answers to the post experiment questionnaire. As the variables did not follow a normal distribution (*p* < 0.05 for all tested variables), we used non-parametric tests to assess the impact of avatar visual fidelity; results are considered significant when *p* < 0.05. Bonferroni's correction was used to adjust alpha values for *post-hoc* pairwise comparisons.

### 4.1. Embodiment and Visual Fidelity

The results of the Friedman Test indicated that there was no significant difference in terms of self-location nor in terms of agency between the three avatar conditions. Nevertheless, agency mean scores are very high (Table 2) for each condition, confirming that visuomotor synchrony is a critical factor contributing to the sense of embodiment in immersive virtual environments.

**Table 2:** Statistical summary of the answers to the post experiment questionnaire presenting each factor of the two dimensions: embodiment (SL, Self-Location; A, Agency; O, Ownership; PE, Proteus effect).

**ID**	**Robot**		**Suit**		**Doppelganger**		***p***
	**$\bar{x}$**	**σ**	**$\bar{x}$**	**σ**	**$\bar{x}$**	**σ**	
SL-Q1	3.26	1.109	3.68	0.976	3.65	1.203	
SL-Q2 (r)	2.74	1.214	2.76	1.232	2.91	1.164	
A-Q1	4.38	0.652	4.41	0.743	4.26	0.828	
A-Q2	4.32	0.684	4.15	0.784	4.26	0.710	
A-Q3	4.15	0.610	4.12	0.808	4.26	0.751	
A-Q4	4.00	0.888	4.00	0.778	4.12	0.669	
O-Q1	2.82	1.141	3.12	0.977	3.59	0.957	0.002
O-Q2 (r)	3.15	1.329	3.56	1.050	3.91	1.138	0.034
O-Q3	2.00	1.181	2.44	1.186	2.76	1.232	< 0.001
O-Q4	3.50	1.187	3.76	1.046	3.82	1.058	0.040
PE-Q1	2.62	1.349	3.12	0.946	2.88	1.149	0.026
PE-Q2	3.35	1.252	3.62	1.155	3.59	1.351	

*Mean and standard deviation are provided for each condition (robot, suit, doppelganger). The level of significance is only provided when p < 0.05. (r) indicates reverse-scored items*.

Friedman Tests indicated significant differences in ownership scores for the four items of the questionnaire: O-Q1 [χ^2^ (2, *N* = 34) = 12.437, *p* = 0.002), O-Q2 (χ^2^ (2 *N* = 34) = 6.747, *p* = 0.034), O-Q3 (χ^2^ (2, *N* = 34) = 13.674, *p* = 0.001), O-Q4 (χ^2^ (2, *N* = 34) = 6.462, *p* = 0.040]. Inspection of the median values showed an increase in ownership from the robot condition to the suit one and a further increase for the doppelganger for the four ownership items.

*Post-hoc* Wilcoxon Signed Rank Tests did not reveal significant differences in the ownership scores between the robot and the suit conditions due to Bonferroni adjusted alpha value used to control type 1 error. However we observed some trends in favor of the suit for O-Q2 (*Z* = 1.988, *p* = 0.047) and O-Q3 (*Z* = 2.064, *p* = 0.039). These tests also indicated that there were significant differences in the ownership scores between the robot and the doppelganger for the first three items in favor of the doppelganger condition: O-Q1 (*Z* = 3.180, *p* = 0.001), O-Q2 (*Z* = 2.764, *p* = 0.006), O-Q3 (*Z* = 3.353, *p* = 0.001). Furthermore, a trend was observed for the last item: O-Q4 (*Z* = 19.945, *p* = 0.052). Wilcoxon Signed Rank Tests also revealed a significant difference in the ownership scores between the suit and the doppelganger for the first item in favor of the doppelganger condition: O-Q1 (*Z* = 2.557, *p* = 0.011) and a trend for the second item: O-Q2 (*Z* = 1.854, *p* = 0.064).

**Figure 6:**
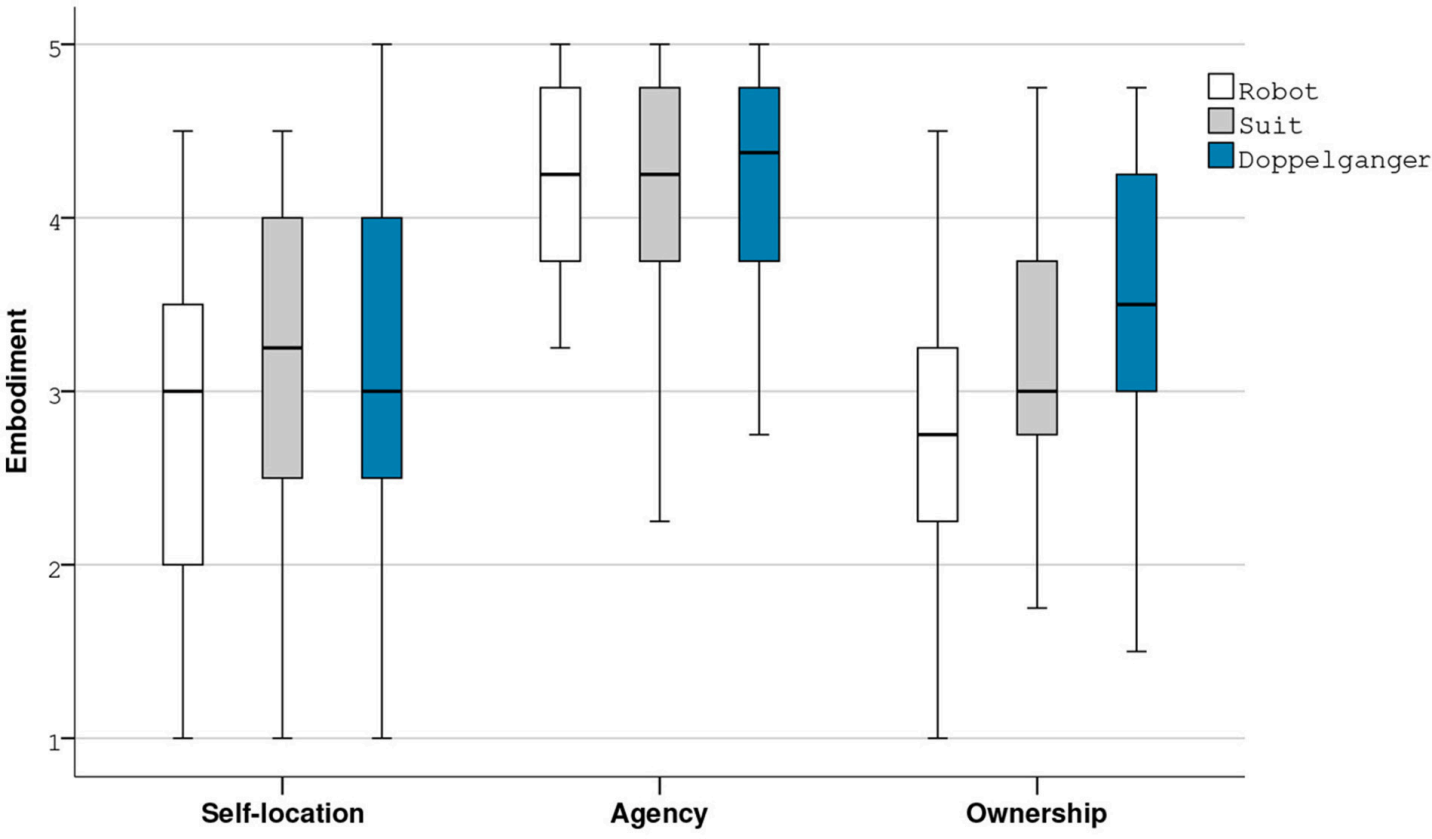
Boxplot of the averages of the SL, A, and O factors of the embodiment dimension.

Taken together our results concerning embodiment in immersive virtual environments highlight an impact of avatar visual fidelity, especially truthfulness, on the sense of ownership (Figure 6), providing evidences of the validity of our first hypothesis. Indeed, our results revealed significant differences especially between the two extreme conditions (robot vs. doppelganger). Furthermore, the observed trends between the suit and the robot or the doppelganger could probably be confirmed with an extended panel (*N* > 34).

### 4.2. Behavioral Data

**Figure 7:**
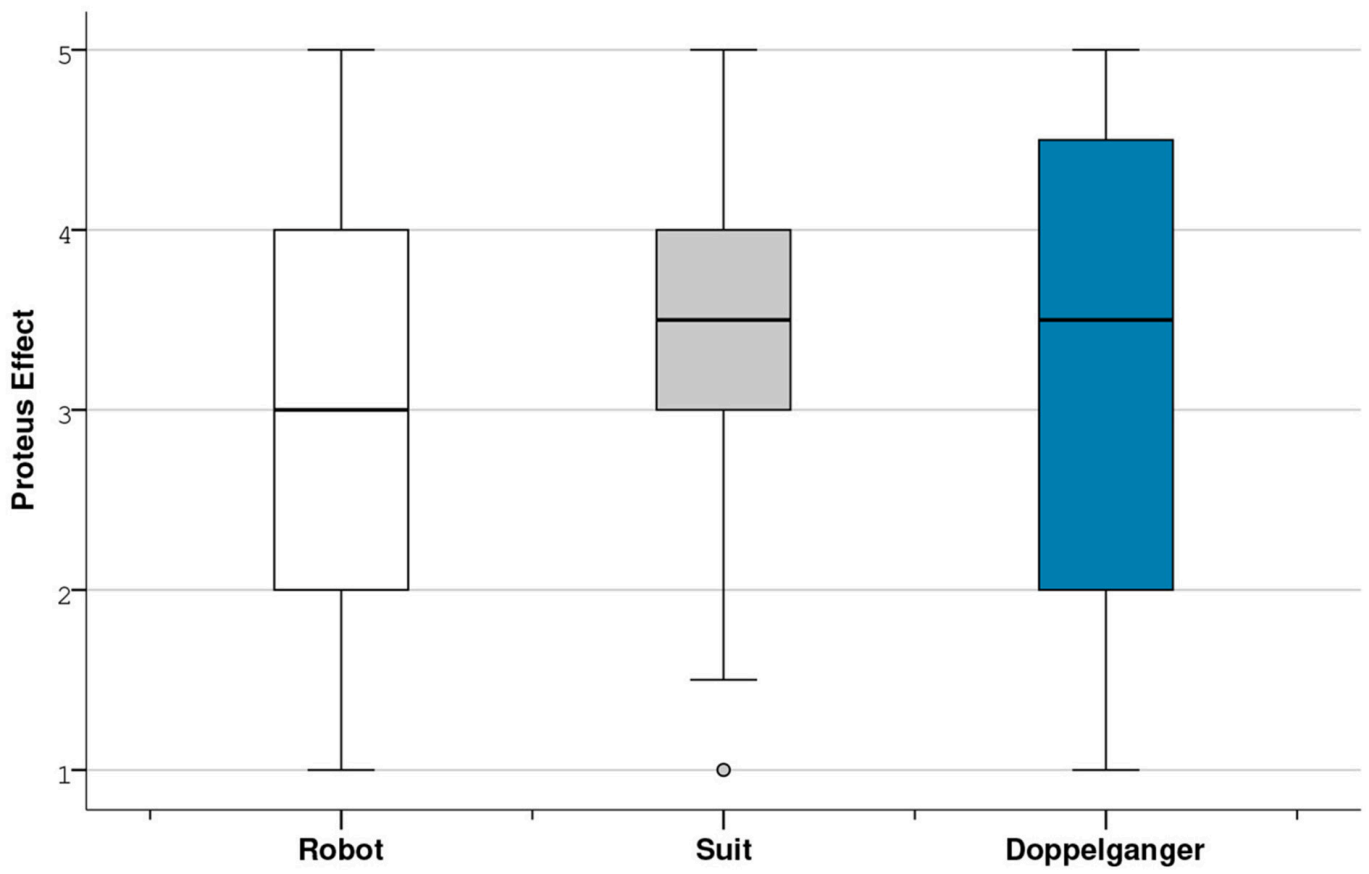
Boxplot of the averages of the Proteus Effect dimension.

A Friedman Test revealed a significant difference for the first question assessing the extent the participants feel vulnerable during the different tasks (PE-Q1) [χ^2^ (2, *N* = 34) = 7.298, *p* = 0.026]. However, *post-hoc* tests with adjusted alpha value did not indicate significant differences in pairwise comparisons. It should be noted that the dispersion of the scores concerning the Proteus Effect is very important (Figure 7). It suggests that each participant could experience the situations differently when their avatar can be threaten.

**Figure 8:**
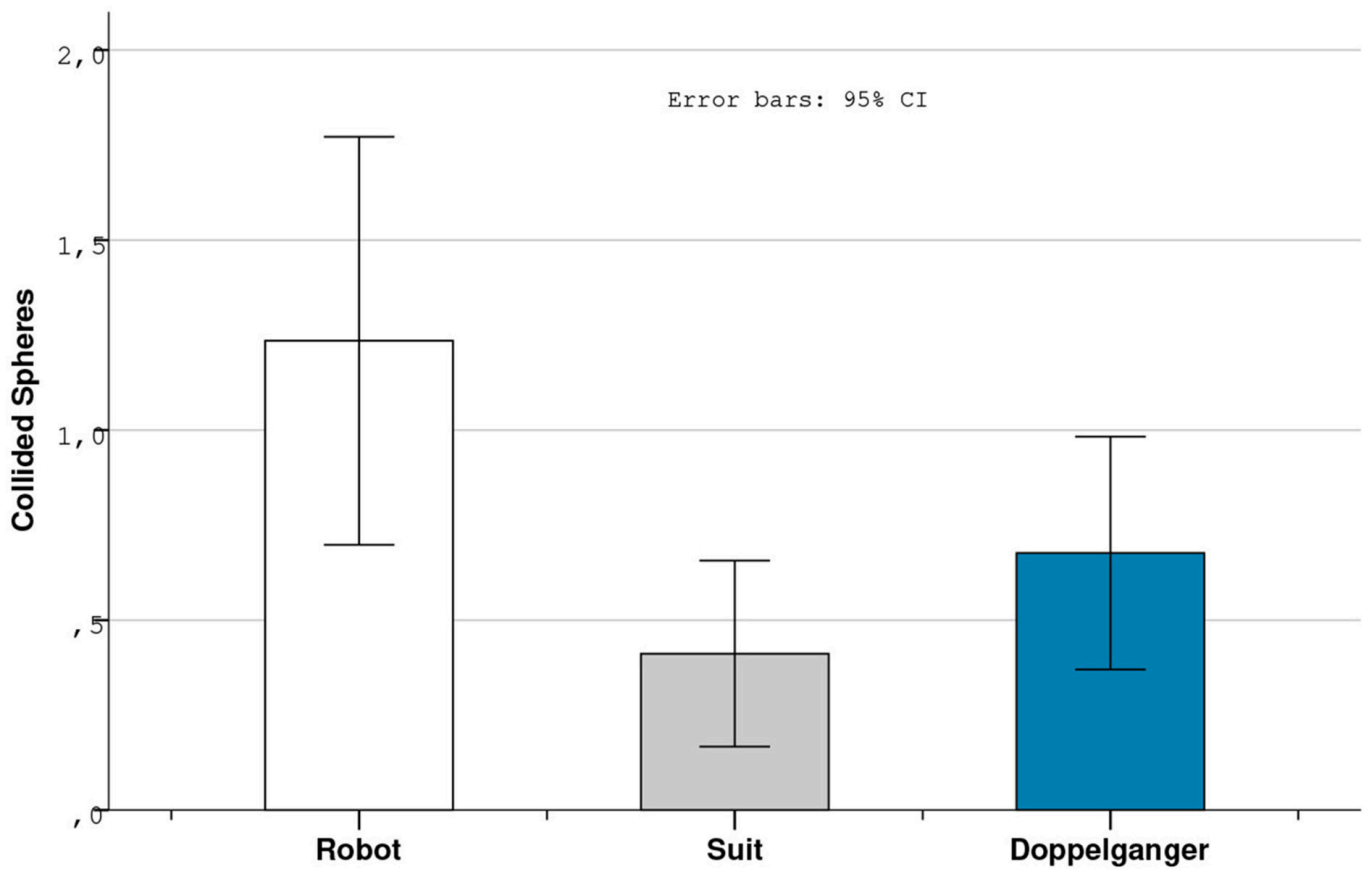
Bars of means of the number of collided spheres.

Concerning objective results based on the data collected by the application, while most of the measured variables did not revealed significant differences, a Friedman Test indicated a significant difference concerning the number of collided spheres [χ^2^ (2, *N* = 34) = 6.024, *p* = 0.049]. Wilcoxon Signed Rank Test revealed a significant difference between the robot and the suit condition with more recorded collisions for the robot (*Z* = 2.328, *p* = 0.020) (Figure 8).

We investigated further these issues during the interviews where participants provided more insights. It appears that half of the panel (*N* = 17) reported adopting different strategies depending on the controlled character. Of those participants, 12 felt more secure using the robot due to its mechanical appearance increasing disconnection from reality. Furthermore, 12 of those 17 subjects also underline the fact that controlling their doppelganger induced more realistic reactions in order to preserve their integrity. These results seem to demonstrate that the subjective experience lived by the participants was affected by avatar visual fidelity, but looking at objective results we cannot confirm that it affected users' performance. We discuss further the reported subjective information and their implications concerning participants' behavior in the next section.

## 5. Discussion

### 5.1. Impact of Avatar Truthfulness on the Sense of Embodiment

Previous work demonstrated that multi-sensory integration like congruent visuomotor synchrony and visuotactile stimulations operate as critical bottom-up factors allowing users to embody different avatars in immersive virtual environments (Kokkinara and Slater, 2014). Our study involving a full-body tracking system acknowledges that some factors of the embodiment process like agency are very high under these conditions. On the other hand, the main focus of our experiment concerns the impact of the top-down factor that is visual fidelity. We used three avatars presenting different levels of truthfulness to evaluate the impact of similarity between users and avatars on the sense of embodiment. Our results confirm our first hypothesis and demonstrate that truthfulness positively impacts the sense of embodiment in virtual environments and especially the ownership factor. Based on complex stimuli in an immersive gaming scenario, our study corroborates and contributes to extend previous findings observed with first-person perspective (Waltemate et al., 2018) also for third-person perspective. Participants of the experiment reported being more connected with their own representation and appreciated having a personalized character:

“*Since he represents me, I think immersion is stronger. You really have the impression to control your character, but it's you.”*

“*I preferred the avatar representing me with my face, for the personification. It's the personalized experience […]. I was more in the skin of my character, the impact was stronger.”*

“*At first I really thought it was my body.”*

However, as expected, some subjects noticed minor issues on their 3D model due to current limitations of scanning technology. According to the Uncanny Valley theory, these inconsistencies could induce revulsion responses of the subjects toward their self-representation. Indeed, due to their high level of realism, participants easily noticed the mismatches between their real face and their virtual one:

“*I felt like I was looking at some kind of copy of myself that wasn't really me.”*

“*The doppelganger creeped me out a little bit, it was a little unwholesome…”*

While this side effect has to be considered before developing virtual reality applications involving avatars based on users' appearance, our results contribute to extend the knowledge concerning the impact of avatar truthfulness. Moreover, they corroborate previous findings demonstrating that avatar visual fidelity can improve the sense of embodiment in immersive virtual environments (Waltemate et al., 2018). Furthermore, Argelaguet et al. (2016) and Latoschik et al. (2017) demonstrate that the levels of anthropomorphism and realism of a virtual character positively influence the sense of ownership. Considering these results together, it is now possible to confirm that the three criteria of avatar visual fidelity (Garau, 2003) (anthropomorphism, realism and truthfulness) affect the sense of embodiment in virtual reality applications based on both first- and third-person perspectives.

### 5.2. Implications of Avatar Visual Fidelity on Users' Behavior

According to the Proteus Effect theory (Yee and Bailenson, 2007; Yee et al., 2009), avatar appearance can affect users' behavior. Our results corroborate partially these findings. Indeed, subjective data collected through the post-experiment questionnaire and the interviews demonstrate that user experience is affected by avatars' appearance as in previous study (Lucas et al., 2016) but few objective data allow to confirm that users' performance is significantly impacted by the controlled character. Nonetheless, half of the participants consider that their avatar influenced their reactions and behavior during the different tasks of the experiment. Many participants reported feeling more secure using the robot thanks to its mechanical constitution:

“*I felt much more powerful with the robot. I found I walked faster on the platforms, I died fewer times. Yeah, I was more confident.”*

“*At first I really thought it was my body, […], the dangers in the experience affected me. I was more in a situation where I had to risk my life. With the robot I rather tried to block the laser, push the balls away. I wouldn't necessarily have tried it with my own character, […], you only have one life.”*

“*For me, playing a game or live a VR experience consists in getting out of the world in which we usually are. That's what I like when I do this kind of experience and it's like there's interference from the physical world in the virtual world and it bothers me a bit.”*

In the light of these insights, using abstract representations can lead to increase disconnection from reality and allows the participants to act with few restraints in the virtual world. On the other hand, the doppelganger condition seemed to induce more realistic behaviors and a kind of preservation instinct. As expected, the participants were less deindividuated, which is one of the requirements to impact users behavior as demonstrated in previous experiments assessing the Proteus Effect (Kilteni et al., 2013; Lugrin et al., 2016). They reported being more careful in order to ensure the integrity of their avatar:

“*At the beginning it put me more in the character and I told myself: "OK, I must not fall because it is me and it scares me", but I was less in the fun. You're really more serious, you have to be careful.”*

“*When I controlled my own body, I really didn't want to be touched. With the robot it might have bothered me a little less. There was less the feeling of life.”*

The participants' comments relate the impact avatars' appearance on their behavior. Although these subjective feedback are only partially in line with our objective data, we tried to assess the impact of avatar visual fidelity on user experience and not for performance purpose. That's why in our context we consider that users' feeling takes precedence over objective observations. Taken together, our results concerning the impact of truthfulness on users' behavior revealed that each type of avatar could be used to produce specific and expected reactions. Doppelgangers seem to induce more serious attitudes and encourage the participants to preserve the integrity of their avatar, self-representations remaining an anchor in the real world. On the other side, more abstract representations allow users to be completely disconnected from reality and to live new experiences.

### 5.3. Limitations

One of the main limitations of our experiment concerns the 3D reconstruction of participants with long hair and relatively thin strands, thus limiting the gender mix of our panel. It should be noted that recent work aims to reproduce complex and optimized hairs for real-time 3D rendering (Hu et al., 2017). In addition, we are currently working on research investigating the impact of gender on the sense of embodiment in immersive virtual environments.

Another limitation of our study concerns the virtual environment we developed for this experiment. Indeed, while the science fiction universe ensures the coherence between the avatars and the environment, it can affect users' experience and therefore the outcomes of the study. To limit this phenomenon, we asked the participants to rate their level of affinity with this kind of universe. The high overall evaluations limit the revulsion responses toward our experiment, but it can also potentially strengthen the measured factors.

## 6. Conclusion

The study reported here investigates the impact of avatar visual fidelity on the sense of embodiment and users' behavior in virtual reality applications relying on a third-person perspective. According to our literature review, avatar visual fidelity could be divided into three factors: anthropomorphism, realism and truthfulness. Our experiment especially focuses on truthfulness, that is to say the similarity between users and avatars.

Our results demonstrated that the sense of embodiment is higher for the participants controlling their self-representation than with more abstract representations like the robot or the suit. As expected, visual fidelity does not seem to impact both self-location and agency. However, our statistical analysis revealed significant differences in terms of ownership between the robot and the doppelganger in favor of the latter. Furthermore, strong trends between the suit and the other conditions were observed and could probably be confirmed with an extended panel. It should be noted that if doppelganger could be used as an embodiment vector, some participants noticed reconstruction flaws on their self-representation reducing their affinity toward their avatar. While it concerns a minority of subjects, it remains necessary to consider current hardware limitations before planning to develop virtual reality applications based on 3D scanning technologies.

Concerning the impact of avatar appearance on users' behavior, a phenomenon known as the Proteus Effect, we can distinguish the subjective results based on our questionnaire, the post-experiment interviews and the objective data collected by our application. Indeed, objective measures revealed few significant differences concerning users' behavior between the three avatars' truthfulness conditions. However, when interviewing the participants after the experiment, half of the panel reported having different reactions and strategies depending on the controlled character. Abstract representations, especially the robot, allow the users to completely disconnect themselves from reality and induce the adoption of more risky behaviors. On the other hand, self-representations maintain a connection with the physical world and encourage users to ensure the integrity of their own avatar.

To conclude, avatar visual fidelity and its truthfulness factor can be a lever to improve the sense of embodiment in immersive virtual environments. However, as it seems to affect users' subjective experience, it must be used wisely to design virtual reality applications inducing expected and specific behaviors.

## 7. Future Work

Based on this study and in the light of our results, we planned to pursue the investigation concerning the sense of embodiment and the Proteus Effect in immersive virtual environments. Our experiment revealed specific behavior between our extreme conditions, the robot and the doppelganger. We would like to cross these conditions to analyze users' reactions when controlling a character presenting a mechanical body, like our robot, but with their own head. Will their behavior be more affected by their mechanical body or will they focus on the preservation of their self-representation?

## Ethics Statement

The panel recruited for the experiment consists of volunteer students in the frame of their educational program. The non-invasive devices of our virtual reality setup are regularly used by the subjects that we have solicited and are accessible to the general public. The protocol excludes the collection of physiological data and we ensured the anonymity of the participants.

## Author Contributions

GG, OC, SH, and SR conceived the study and the experimental protocol. GG developed the VR application. GG and OC ran the experiment and analyzed the objective and subjective data. GG, OC, and SH wrote the paper.

### Conflict of Interest Statement

The authors declare that the research was conducted in the absence of any commercial or financial relationships that could be construed as a potential conflict of interest.
